# 

**Published:** 1907-06-15

**Authors:** 


### CONTENTS

Event and Comment - - - - - - - - - - - - 269
Method of Retaining Teeth After Regulating, - - *By W. S. Locke, D.D.S.* 276
Report of Ohio State Dental Society Meeting.—President's Address - - 283
Abuses of Amalgams and Their Treatment, - - *By N. K. Garhart* 285
Dental Army Service in the Philippines, *By S. D. Boak, U. S. A. Dental Surgeon* 289
Oral Prophylaxis - - - - *By W. H. Van Deman, D.D.Sc.* 296
Some Practical Points About Porcelain, - *By E. H. Shannon, D.D.S.* 298
Some Practical Suggestions, - - - - *By E. C. Sherman, D.D.S.* 299
Mechanical Method for Correction of Facial Expression, *By Weston A. Price, D.D.S.* 300
Gold Inlays, - - - - - - - - *By T. P. Hinman, D.D.S.* 301
Clinics at the Ohio State Meeting.—A Crown Whose Band Remains Invisible, *By S. D. Ruggles, D.D.S.* 306
The Use of Flexible Rubber in the Retention of Artificial Dentures after Other Means have Failed, - - - - - *By Newell H. Grove* 307
A Bandless Richmond Crown, - - - - *By J. C. Longfellow* 308
Combination Gold-Faced Platinum Crown with Porcelain Cush and Pace, *By Stanton A Stuart* 310
Slipjoint Attachment to Handpiece for Moistening Grindstone, *By David Stern, B.S., D.D.S.* 311
A Method of Making a Gold Inlay, - - - *By Henry Barnes, M.D.* 312
A Base for a Richmond Crown, - - *By James E. Boyd, D.D.S.* 313
Obituary - - - - - - - - - - - - - - - 315
Indents - - - - - - - - - - - - - - - - 316
Announcements - - - - - - - - - - - - - - 318

